# Survival of Infants Born to HIV-Positive Mothers, by Feeding Modality, in Rakai, Uganda

**DOI:** 10.1371/journal.pone.0003877

**Published:** 2008-12-09

**Authors:** Joseph Kagaayi, Ronald H. Gray, Heena Brahmbhatt, Godfrey Kigozi, Fred Nalugoda, Fred Wabwire-Mangen, David Serwadda, Nelson Sewankambo, Veronica Ddungu, Darix Ssebagala, Joseph Sekasanvu, Grace Kigozi, Fredrick Makumbi, Noah Kiwanuka, Tom Lutalo, Steven J. Reynolds, Maria J. Wawer

**Affiliations:** 1 Department of Clinical Research Studies, Rakai Health Sciences Program, Entebbe, Uganda; 2 Department of Population, Family and Reproductive Health, Johns Hopkins University Bloomberg School of Public Health, Baltimore, Maryland, United States of America; 3 Department of Epidemiology and Biostatistics, Makerere University School of Public Health, Kampala, Uganda; 4 Department of Disease Control, Makerere University School of Public Health, Kampala, Uganda; 5 Department of Medicine, Clinical Epidemiology Unit, Makerere University School of Medicine, Kampala, Uganda; 6 Department of Biostatistics and Data Management, Rakai Health Sciences Program, Entebbe, Uganda; 7 Department of Quality Control and Quality Assurance, Rakai Health Sciences Program, Entebbe, Uganda; 8 Division of infectious Diseases, School of Medicine, Johns Hopkins University, Baltimore, Maryland, United States of America; 9 Laboratory of Immunoregulation, Division of Intramural Research, National Institutes of Allergy and Infectious Diseases, National Institutes of Health, Bethesda, Maryland, United States of America; Institute of Clinical Effectiveness and Health Policy, Argentina

## Abstract

**Background:**

Data comparing survival of formula-fed to breast-fed infants in programmatic settings are limited. We compared mortality and HIV-free of breast and formula-fed infants born to HIV-positive mothers in a program in rural, Rakai District Uganda.

**Methodology/Principal Findings:**

One hundred eighty two infants born to HIV-positive mothers were followed at one, six and twelve months postpartum. Mothers were given infant-feeding counseling and allowed to make informed choices as to whether to formula-feed or breast-feed. Eligible mothers and infants received antiretroviral therapy (ART) if indicated. Mothers and their newborns received prophylaxis for prevention of mother-to-child HIV transmission (pMTCT) if they were not receiving ART. Infant HIV infection was detected by PCR (Roche Amplicor 1.5) during the follow-up visits. Kaplan Meier time-to-event methods were used to compare mortality and HIV-free survival. The adjusted hazard ratio (Adjusted HR) of infant HIV-free survival was estimated by Cox regression. Seventy-five infants (41%) were formula-fed while 107 (59%) were breast-fed. Exclusive breast-feeding was practiced by only 25% of breast-feeding women at one month postpartum. The cumulative 12-month probability of infant mortality was 18% (95% CI = 11%–29%) among the formula-fed compared to 3% (95% CI = 1%–9%) among the breast-fed infants (unadjusted hazard ratio (HR)  = 6.1(95% CI = 1.7–21.4, P-value<0.01). There were no statistically significant differentials in HIV-free survival by feeding choice (86% in the formula-fed compared to 96% in breast-fed group (Adjusted RH = 2.8[95%CI = 0.67–11.7, P-value = 0.16]

**Conclusions/Significance:**

Formula-feeding was associated with a higher risk of infant mortality than breastfeeding in this rural population. Our findings suggest that formula-feeding should be discouraged in similar African settings.

## Introduction

In resource-limited settings with high prevalence of HIV, health workers face a dilemma on how best to advise HIV-infected mothers on infant-feeding practices. Current WHO/UNAIDS guidelines recommend formula feeding as long as it is “acceptable, feasible, affordable, sustainable and safe” [1]. Most programs have tried to ensure safety through education of mothers on hygienic preparation and storage of formula, but the extent to which such education can ensure safety is unknown. Several studies have shown excess mortality and/or morbidity among formula-fed infants compared to breast-fed infants [2]–[4]. Although some studies found comparable HIV-free survival between feeding methods [4], others showed better HIV-free survival among the formula-fed infants, thus making a case for formula-feeding for prevention of breast milk transmission of HIV [5]–[6].

However, most studies were conducted in urban settings with access to piped water and to health care, and were secondary analyses of clinic-based randomized trials. These study settings may not be generalizable to rural populations where a majority of people in Africa reside. We present findings from an evaluation of a community-based service program for prevention of mother-to-child HIV transmission (pMTCT) in rural Rakai district of Uganda, where HIV-infected women were offered free formula as an alternative to breast-feeding(if they freely chose not to breast-feed), following infant-feeding counseling for prevention of breast milk transmission of HIV. Breast-feeding is the normative practice in this population regardless of maternal HIV-status. We compared infant mortality and HIV-free survival between formula-fed and breast-fed infants born to HIV-infected mothers.

## Methods

### Study populations and procedures

The objective of this programmatic evaluation was to compare mortality and HIV-free survival among formula-fed and breast fed infants born to HIV-infected mothers, who self-selected their preferred method of infant feeding following counseling on prevention of breast milk HIV transmission. The data were derived from the ARV-related Maternal-Infant Study (ARMIS)-an offshoot of the Rakai Community Cohort Study (RCCS). The RCCS maintains annual HIV surveillance in a cohort of over 12,000 adults aged 15–49 in 50 villages in rural Rakai district. The cohort monitored HIV/STD incidence and prevalence as well associated behavioral and demographic determinants. Household demographic data were collected though an annual census and included information on household possessions (used as a proxy for social-economic status), access to water and electricity, and possession of sanitary facilities such as toilets/latrines. During the annual surveys women in this cohort were also screened for pregnancy by menstrual history, physical examination, and urinary hCG pregnancy tests. Consenting cohort participants were screened for HIV infection at each annual visit.

From 2005, all consenting pregnant HIV-infected mothers from RCCS, and their newly born infants, were enrolled in a separate cohort study called “ARV-related Maternal-Infant Study (ARMIS)”, which assessed the effects of availability of antiretroviral therapy (ART) on health outcomes of pregnant women and their infants. Pregnant HIV-positive women identified through RCCS were visited at home by midwives who were also trained HIV-counselors (“midwife counselors or MWCs”).

Pregnant women were asked to notify the MWCs (by cell phone or through a messenger) as soon as they had delivered. Mother-infant pairs were followed-up at home by the MWCs soon after notification of birth (usually within forty eight hours), then at one, six and twelve months after birth. Structured questionnaires were used to collect information on maternal and infant-morbidity and mortality as well as infant feeding. Heel-prick infant blood was collected for HIV PCR at all follow-up visits starting at one month. Infant HIV was not determined at birth. Mothers were offered their infant's HIV results and counseling on care and infant feeding.

Pregnant women were offered prenatal voluntary HIV counseling and testing (VCT), antenatal care, hematinics, multivitamins, and presumptive malaria prophylaxis using Fansidar (Sulphamethoxazole + pyrimethamine) as recommended by the Ugandan Ministry of Health (MOH). They were also offered the single dose Nevirapine (sdNVP) for pMTCT using procedures described previously [7]. After September 2007, prophylaxis was changed to a combination antiretroviral regimen of AZT starting at 28 weeks gestation, in addition to sdNVP and 3TC given at the start of labor with a 7-day tail of 3TC and AZT postpartum for the mothers, and AZT syrup for the infant as per WHO recommendation [8].

Women who had WHO clinical stage 4 disease or CD4 counts less than or equal to 250 cells/ul were offered free antiretroviral therapy (ART) through a community-based program. They also received basic HIV care that included routine cotrimoxazole prophylaxis, treatment of opportunistic infections, insecticide treated bed nets for prevention of malaria and safe water vessels with hypochlorite for prevention of intestinal infections.

HIV-infected infants were offered cotrimoxazole prophylaxis starting at six weeks and ART if they met the WHO eligibility criteria for initiation of antiretroviral therapy.

Mothers and their Infants were symptomatically treated at home for simple health problems or referred to the government health units for follow-up care and for treatment that could not be provided by the MWCs at home.

Government and private health care facilities are available in Rakai, but services are limited and most clinics accessible to rural communities (<5 Km) are staffed by non-physicians. Services are frequently interrupted by absence of personnel and stock outages of essential medicines.

As part of the pMTCT service program, mothers were given infant feeding counseling for prevention of breast milk HIV transmission by the MWCs at the pre-natal visit and then provided follow-up counseling at other scheduled study visits. They were then allowed to make an informed choice between breast-feeding or to use the free formula (NAN from Nestle) provided by the pMTCT service program. Mothers who chose to breast-feed, were encouraged to breast-feed exclusively for six months and then wean their infants thereafter. Exclusive breast feeding was defined as breast feeding with no added supplements, and mixed breast-feeding was defined as breast-feeding with additional supplementary feeds (excluding medications). Mothers who chose to use formula were trained in hygienic preparation of feeds, measurement of the correct amounts of formula appropriate for the child's age and were provided with a free cup, spoon as well as a vacuum thermos flask for storage of night feeds. Mothers were discouraged from using infant feeding bottles as these were deemed difficult to keep clean. Mothers were encouraged to wash utensils with soap and water and to boil the utensils after washing. Re-use of feeding utensils without washing was strongly discouraged. Mothers were asked to only use freshly prepared formula and to discard any leftover.

During the consent process for ARMIS, women were informed that their service-based data could be linked to data they provided through ARMIS. Therefore all women used for this evaluation provided consent for use of their data from the pMTCT service program.

The RCCS and ARMIS were approved by the Science and Ethics Committee of Uganda Virus Research Institute, the Uganda National Council of Science and Technology and US-based Western IRB

### Statistical analysis

Only infants alive at birth were included in this analysis. In the case of multiple births, only the first born twin was included. The feeding option practiced by mothers at the first postpartum visit (usually within 48 hours after birth) was used to classify infants as either breast-feeding or formula-feeding.

Maternal and Infant baseline characteristics were compared between the feeding groups. The Mann-Whitney U test was used for continuous variables while Fischer's exact test was used for categorical variable. Infant mortality and the composite outcome of mortality or HIV infection (i.e., the complement of HIV-free survival) in the two feeding groups were compared using Kaplan-Meier time-to-event methods. Multivariable Cox proportional hazards regression was used to estimate adjusted hazard ratios (adj HR) and 95% confidence intervals (95%CI) of infant mortality or HIV-infection, adjusting for maternal age, and use of ART as a time-varying covariate. CD4 counts were available for 84% of formula feeding mothers and only 54% of breast feeding mothers, so adjustment for maternal CD4 counts omitted many observations and resulted in poor model fit. However, receipt of ART was contingent on a CD4 count <250 so low CD4 counts were strongly negatively correlated with receipt of ART, and models adjusting for ART provided a better model fit. Therefore, in the final model, receipt of ART was used for adjustment instead of CD4 counts.

Censoring for the mortality outcome occurred due to loss to follow-up at the visit when this was first noted. For the composite outcome of mortality and HIV-infection, censoring occurred due to loss to follow-up and absence of an HIV-result. Because we could not distinguish between in utero/peripartum HIV-infection and early breast-milk transmission at one month, all mother-child pairs in which transmission was detected at one month were left censored. One observation with a missing HIV test at one month where the first HIV-positive result was detected at six was also censored.

The proportional hazards assumption was tested using Schoenfeld and scaled Schoenfeld residuals[9]. Model goodness-of-fit was tested using graphical methods based on Cox-Snell residuals[10].

Statistical analysis used STATA software (Release 9.2. Stata Corporation, College Station, Texas, USA).

## Results

One hundred and eighty seven (187) infants, including 10 twins, were born alive to the HIV-positive mothers. After excluding the second-born twins, 182 infants were included in this analysis. Seventy five (41%) of mothers chose to formula-feed and 107 (59%) mothers chose to breast-feed.

Table 1 shows baseline maternal and infant characteristics. Mothers who practiced formula-feeding were significantly older, had higher parity, and were more likely to have received ART or pMTCT drugs (92.0%) compared to breast feeding mothers (73.0%, P-value<0.01). Mothers who used formula also had lower CD4+ T cell counts (412 cells/mm^3^ than breast-feeding mothers (606 cells/mm^3^,P-value <0.01). There were no differences between the feeding groups with respect to education or access to protected water supplies. There were no differentials between the infants in both groups with respect to gender, birth weight and prematurity.

**Table 1:** Baseline Maternal and Infant Characteristics.

	Formula N = 75	Breast-fed N = 107	P-value
**Maternal Characteristics**			
Age(mean, SD)	29.3(5.3)	26.1(5.4)	<0.01
Marital status—n(%)			
Married	53(70.7)	77(72.0)	0.87
Education–n(%)			
Primary/No education	35 (46.7)	46 (43.0)	
Post-primary	40 (53.3)	61 (57.0)	0.65
Mean Parity (SD)	4.3(2.2)	3.4 (2.1)	<0.01
Protected water source*	56(74.7)	73 (68.2)	0.41
Mother on ART before birth	20(26.7)	1(0.9)	<0.01
NVP/AZT prophylaxis or ART	69(92.0)	78(73.0)	<0.01
CD4 counts/ul–Mean(SD)**	412(276)	606(254)	<0.01
**Infant Characteristics**			
Birth weight (SD)	3.1(0.5)	3.1(0.6)	0.92
Preterm birth n(%)	11 (14.7)	19 (17.7)	0.69
Given NVP/AZT syrup for PMTCT	72 (96.0)	90(84.1)	<0.01

* Safe water source defined to include boreholes, protected spring or tap water.

** 61 missing baseline CD4 count

**Figure 1:**
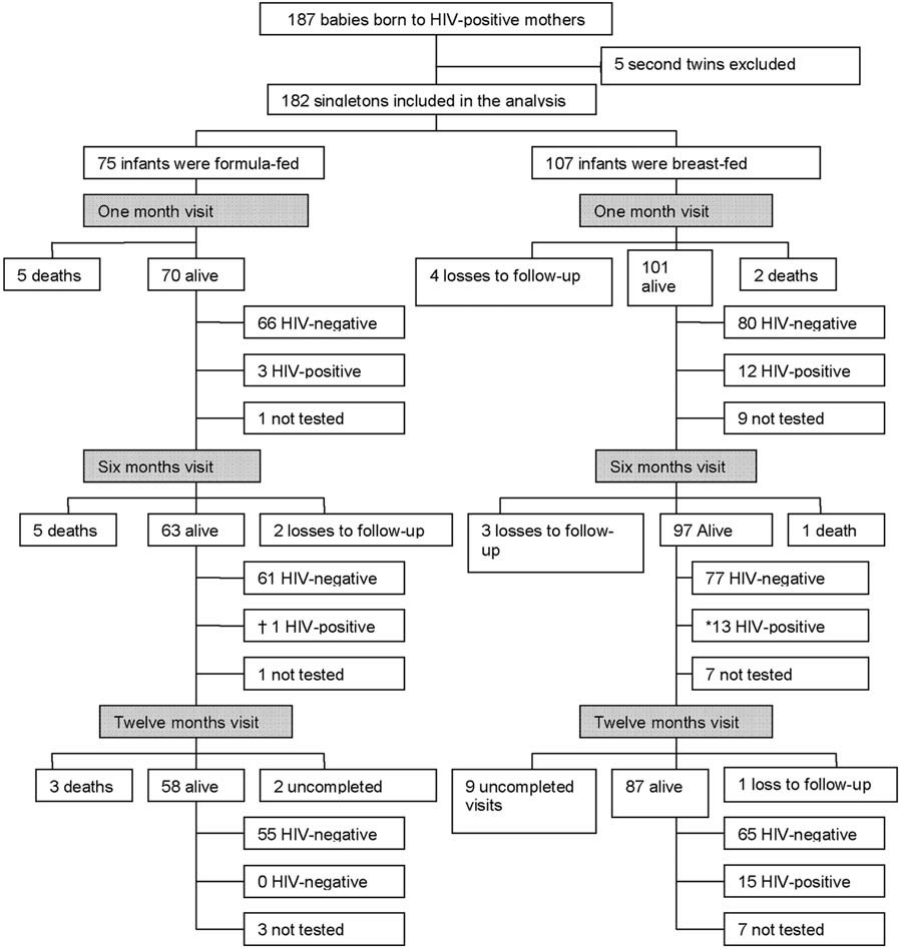
Consort diagram showing follow-up of infants, losses to follow-up, deaths and HIV-infection. * Includes one new HIV-infection observed at six months for an infant who was not tested at one month. † Infection occurred by one month. Failure to be tested was a result of refusal by the mothers to have their babies tested and a minority of cases it was due to insufficiency of the infant sample.

Figure 1 is a consort diagram showing the follow-up of infants. Cumulative follow-up (including deaths) rates were similar among formula-fed and breast-fed infants at all visits (100% vs. 96 %, P-value = 0.09 at one month, 97% vs. 93%, P-value = 0.23 at six months and 93% vs. 83%, P-value = 0.06 at twelve months).

None of mothers who chose to use formula reported any breast-feeding at all subsequent visits. Among the breast-fed infants, all infants seen alive at one month were still breast-feeding. The proportions breast-fed among surviving infants were 85% (82/97) at six months and 70% (61/87) at twelve months. However, exclusive breast-feeding was practiced by only 25% (25/101) of lactating women seen at one month and by 18% (17/97) at six months. The major infant feeds in addition to breast milk used by non-exclusive breast-feeding mothers at one month consisted of cow's milk (25%), mushroom soup (65%), and water (25%). At six months, the additional infant feeds were cow's milk (83%), mushroom soup (58%), water (40%), maize porridge (56%), millet porridge (41%), soya porridge (5%) and solid feeds (56%). The major feeds offered to infants whose mothers weaned them by six months included cow's milk (88%), mushroom soup (59%), millet porridge (88%), maize porridge (59%) and solid feeds (53%).

### Infant mortality, HIV-infection and infant HIV-free survival

The proportion of HIV infected infants at one month were 13.0% (12/92) among the breast-fed compared to 4.4% (3/69) among the formula-fed infants (P-value = 0.06)

**Figure 2:**
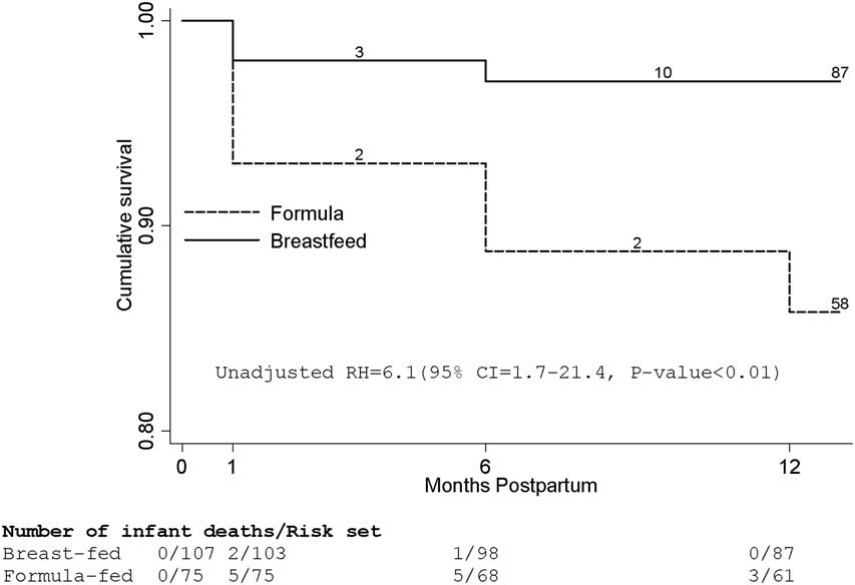
Kaplan-Meier cumulative probabilities of survival from death by feeding group. Actual visits grouped by the three scheduled visits at one, six and twelve months after birth.

Figure 2 shows the Kaplan-Meier cumulative probabilities of survival from death. The 12-month cumulative probability of death was 18% (95% CI = 11%–29%) among the formula-fed compared to 3% (95% CI = 1%–9%) among the breast-fed (unadj. HR = 6.1(95% CI, 1.7–21.4, P-value<0.01). We only present unadjusted analysis for this mortality endpoint since infant HIV-testing started at one month and we could not differentiate between in utero/peripartum transmission and early breast milk transmission between birth and one month, so adjusting for infant HIV-infection when analyzing the mortality endpoint would lead to censoring of the seven failure events that occurred between birth and one month prior to HIV testing. Since some infants in the breast-feeding group were weaned before twelve months we explored whether their exclusion would affect the results. The hazard ratio of mortality comparing formula-fed to breast-fed after exclusion of infants weaned before twelve months was 6.3 (95% CI = 1.4–28.0, P-value = 0.02)

All the three infants infected with HIV by one month of age in the formula-feeding group died by twelve months, whereas no deaths occurred among the twelve infants infected with HIV at one month in the breast-feeding group. The hazard ratio after exclusion of the HIV-infected formula fed infants was 4.9 (95% CI =  1.3–17.6, P = 0.02).

**Figure 3:**
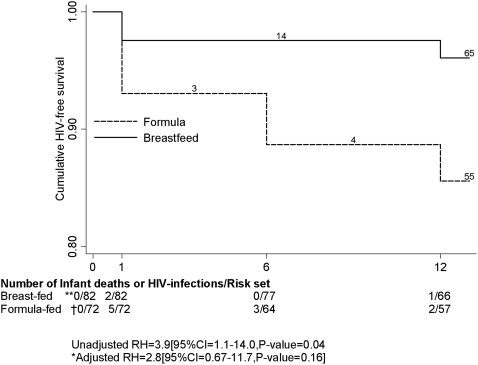
Kaplan-Meier cumulative probabilities of HIV-free survival by feeding group. Actual visits grouped by the three scheduled visits at one month, six months and twelve months after birth. *Adjusted for maternal age and maternal antiretroviral therapy. ** Twenty five observations left-censored for the following reasons: 12 babies were HIV-positive at one month without HIV results at birth, 9 were not tested for HIV at one month, and 4 losses to follow-up. † Three infants who were HIV-positive at one month were left-censored.

Figure 3 shows the Kaplan-Meier cumulative probabilities of HIV-free survival. The 12-month cumulative probability of infant HIV infection or death was 14% (95% CI 8%–25%) in the formula-fed infants and 8% (95% CI = 4%.–16%) among the breast-fed infants (unadjusted HR = 3.9 [95%CI 1.1–14.0, P-value = 0.04), and adjusted HR  = 2.8 [95%CI 0.67–11.7,] after adjustment for maternal age and maternal ART use. The corresponding infant HIV-free survival at twelve months was 86% in the formula group compared to 96%.among the breast-fed (P-value = 0.16).

### Post-evaluation decisions

After a review by the Study investigators confirmed the excess mortality in non-breastfed infants, further provision of formula in the pMTCT service program was stopped on 6^th^ August 2007 for newly delivered babies.

The pMTCT program then decided to encourage exclusive breast-feeding for the first six months of life followed by gradual introduction of complementary feeds (not provided by the program) with continuation of breast-feeding for a duration of the mothers' informed choice. At this time however, ongoing formula-feeding was still being practiced by 29 mothers. Since breast-feeding could not be re-established, these infants continued to formula-feed. Mothers of the 29 infants were re-visited in their homes to evaluate their hygiene practices and to support safe continued use of formula. During the home visits, MWCs observed preparation and storage of formula and completed a household hygiene survey to assess hygienic practices. Monthly visits were planned to support the mothers for the rest of the formula-feeding time.

In the household hygiene survey, all mothers reported that they used soap and water to wash feeding utensils, but 17 (59%) mothers admitted to re-use of feeding utensils without washing. Infant feeding bottles, which were contraindicated by the program, were used by 25 (86.7%) of mothers. Storage of leftover feeds for later use was practiced by 5 (17.2%) of mothers. Overall, 9 (31.1%) of mothers reported difficulty maintaining clean utensils and 19 (65.5%) reported difficulty measuring the correct amount of formula powder. Nine mothers (31.0%) reported difficulty keeping utensils clean for the night feeds. Seventeen mothers (59%) reported having to delegate formula feeding to caretakers. Eight out of 17 (47.1%) of mothers delegated feeding to older children, while the remainder delegated feeding to the father or another adult relative. Twenty-three homes (79.3%) had a toilet facility, but only 11 of the 23 (47.8%) of homes with a toilet had hand washing facilities.

## Discussion

Infant mortality among formula-fed infants born to HIV-infected mothers was over six times higher compared to mortality of breast-fed infants. However, there were no differences in HIV-free survival. Our results are in agreement with earlier studies [2]–[4] conducted mainly among trial participants in urban settings. However, the excess mortality associated with formula feeding in this rural setting is substantially greater than that reported in the earlier urban studies. This suggests that the risk of mortality with formula-feeding could be much greater in rural populations with limited access to clean water and medical care. We believe this is the first evaluation of survival in formula-feeding versus breast-feeding by HIV-infected mothers in a programmatic setting. Such information is important to guide policy on infant feeding by HIV-infected mothers. We found that preparation and storage of safe formula were inadequate despite the counseling and support given by the program. In this rural population, less than 4% of the households had access to tap water; the majority of mothers had to fetch water from communal stand pipes or wells which are unlikely to be safe sources of clean water. Mothers failed to follow guidelines for sterile preparation and storage of formula, for cleansing of utensils and for avoidance of bottle feeds. This poor practice probably led to contamination of formula feeds and consequent gastrointestinal infections in the children.

Our findings also suggest that formula-feeding may be particularly hazardous for HIV-infected infants, since all infants HIV infected by one month of age in the formula-feeding group died by twelve months, compared to none in the breast-feeding group. This excess risk to HIV-infected non-breast-feeding infants has been reported in earlier studies [11], [12]. However, the excess mortality with formula-feeding remained, even after excluding infants infected with HIV-at one month of age, suggesting that formula feeding is deleterious even among HIV-uninfected infants.

This programmatic evaluation has limitations of potential residual confounding, since women self-selected their preferred method of infant feeding. The higher rates of ART use and use of sdNVP prophylaxis by formula feeding mothers (Table 1), suggest that sicker mothers or those who were more health conscious may have opted for non-breastfeeding. The high rates of non-exclusive breast-feeding could have reduced the differential in mortality between the formula and breast fed infants, since non-exclusive breast-feeding is associated with higher rates of HIV transmission and morbidity [13]. Mixed breast feeding was practiced by the majority of mothers, despite efforts to emphasize the importance of exclusive breast feeding, indicating the difficulties mothers encounter in practicing exclusive feeding. Since Rakai is predominantly rural, we had too few women from urban settings to assess whether formula feeding differed between rural and urban communities. Lack of infant HIV-testing at birth reduced our ability to do appropriate multivariate analysis for the mortality outcome and also led to left-censoring of a number of observations for the HIV-free survival outcome.

However, our findings of a six-fold higher mortality risk associated with formula-feeding strongly suggest that formula-feeding should be discouraged in similar rural African settings. There is an urgent need for strategies for prevention of breast milk HIV transmission such as prolonged infant antiretroviral prophylaxis [16], [17] or maternal HAART during lactation [18]. Exclusive breast-feeding with abrupt weaning at six months of age has also been associated with high rates infant morbidity and mortality [13] and WHO has updated guidelines to allow for more prolonged breastfeeding [14]. However, breast milk transmission will continue as long as breast feeding is maintained [15].

In conclusion, we found an excess infant mortality with formula feeding in a rural African population of HIV-infected mothers, which suggests that formula feeding should not be promoted in these settings.
